# Verbena Hastata (Tall Blue Vervain,) and V. Urticifolia, (Common Vervain)

**Published:** 1854-04

**Authors:** Silas Hubbard

**Affiliations:** Buffalo


ART.— *Verbena Hastata* (*Tall Blue Vervain,*) *and V. Urticifolia,* (*Common Vervain.*) By SILAS HUBBARD, M. D.

Having become well satisfied that the roots of the above-named plants possess valuable medical virtues with which the medical profession are not generally acquainted, I am induced to offer a few suggestions in regard to them, trusting that should the experience of others verify the conclusions I have drawn from my own, I shall thereby add something to the general stock of useful knowledge.

These two varieties of vervain possess essentially the same properties. They principally grow on the road-sides, in the vicinity of towns and villages. The verbena urticifolia grows more abundantly, and has a more luxuriant root than the other variety, and is the kind I generally use—I will, therefore, confine myself to it. The root, when fresh, has a peculiar characteristic odor, and a nauseous and very bitter taste. The latter part of September, and the first of October, is the best time to collect the root. I sometimes give it in the form of tincture, but usually in infusion. I prefer to merely pour boiling water on it and let it cool forthwith, because, by simmering or boiling it, some of its qualities are dissipated. The complaints for which I have prescribed it with the most benefit, are the various types of intermittent. I am convinced, by my own experience, that it is fully as efficacious as the best cinchona bark in curing these complaints. To cure an intermittent, administer the infusion of the dried root of the strength of 1 oz. to a pint of water; dose f℥ss every four hours during the apyrexia, and it can be administered with good effect, and without any injurious tendency, even during the paroxysm; given in this way, or with brandy, wine or dilute alcohol, the patient rarely suffers a second attack. Its use ought to be continued for some time after the cure to prevent a relapse. It is not necessary to precede its use with a cathartic or an emetic, as physicians frequently do in giving quinine or bark, because, together with its tonic effect, it also promotes the secretions and acts as an alterative. When the patient is bilious, or his stomach foul, it manifests emetic qualities, and thus cures these complaints by its combined effects. I have frequently given it in remittent fevers of various grades, and in many instances it seemed to cut short the disease. It can be given with advantage even during the fever. I have given it in the various forms mentioned, with good results in jaundice depending on a torpor of the liver, and also simple obstructions of the biliary ducts. In its febrifuge powers it seems to resemble quinine more than any other substance, and in fevers I have often used it as a substitute for that article. It is not so apt to irritate the stomach and bowels, neither is it liable to aggravate a fever when given during the paroxysm, as quinine sometimes does. It slightly promotes diaphoresis, and never checks the cutaneous exhalations, as quinine and many other bitter medicines occasionally do. It never creates faintness and nervous prostration, as quinine sometimes does when given in large and frequent doses. It answers as a good tonic during convalescence from the fevers I have mentioned; also typhus and typhoid fevers, and is very effectual in preventing relapses. While the cold infusion acts as a good general tonic, it is also an excellent remedy for *anorexia* consequent on *intemperance*, and also for simple debility, indigestion, and dyspepsia.

BUFFALO, February, 1854.

